# A Magnetically Coupled Piezoelectric–Electromagnetic Low-Frequency Multidirection Hybrid Energy Harvester

**DOI:** 10.3390/mi13050761

**Published:** 2022-05-11

**Authors:** Yongqiang Zhu, Zhaoyang Zhang, Pingxia Zhang, Yurong Tan

**Affiliations:** School of Mechanical and Automotive Engineering, Qingdao University of Technology, Qingdao 266520, China; zzy18920573109@163.com (Z.Z.); zpx614@163.com (P.Z.); weiwuwenjin@163.com (Y.T.)

**Keywords:** multidirectional vibration energy harvesting, hybrid energy harvester, piezoelectric, electromagnetic, magnetic coupling

## Abstract

The traditional single electromechanical conversion energy harvester can collect energy only in a single vibration direction. Moreover, it requires high environmental vibration frequency, and its output power is low. To solve these problems, a cross-shaped magnetically coupled piezoelectric–electromagnetic hybrid harvester is proposed. The harvester comprised a ring-shaped support frame, a piezoelectric generation structure, and an electromagnetic generation structure. The harvester could simultaneously generate energy piezoelectrically and electrically, in addition, it could generate electricity efficiently at a lower environmental vibration, and it can collect the energy in two vibration directions simultaneously. To verify the effectiveness of the device, we set up a vibration experiment system and conducted comparative experiments about non-magnetically coupled piezoelectric, magnetically coupled piezoelectric, and magnetically coupled piezoelectric–electromagnetic hybrid energy harvesters. The experimental results showed that the output power of the magnetically coupled piezoelectric–electromagnetic hybrid energy harvester was 2.13 mW for the piezoelectric structure and 1.76 mW for the electromagnetic structure under the vibration of single-direction resonant frequency. The total hybrid output power was 3.89 mW. The hybrid harvester could collect vibration energy parallel to the ring in any direction. Furthermore, compared with the non-magnetically coupled piezoelectric energy harvester and the magnetically coupled piezoelectric energy harvester, the output power was increased by 141.6% and 55.6%, respectively.

## 1. Introduction

IoT (Internet of Things) technologies require more sensors and electronic devices, which require wired power or chemical batteries [1,2] to maintain operation. However, wired power sources have problems such as complex wiring, cumbersome laying (especially in bridges and tunnels), and the need for additional protection. Furthermore, conventional chemical batteries have problems of regular replacement, recharging, and environmental pollution [3,4,5]. With this in mind, people have started to search for new energy provision options. The available clean energy sources present in the nature are thermal, solar, wind, vibration, and acoustic energy. Among them, vibration energy is widely available in industry and nature [6,7,8]. There are various types of devices for collecting vibration energy. These can be classified into electromagnetic harvesting devices [9,10,11], electrostatic harvesting devices [12,13,14], piezoelectric harvesting devices [15,16,17], and frictional electric harvesting devices [18,19,20] according to their different principles of power generation. The operating principle of electromagnetic energy harvesting devices is based on Faraday’s law of electromagnetic induction, and their energy recovery devices are simple and do not require separate power supplies [21,22]. Compared with electromagnetic energy harvesting devices, electrostatic energy harvesting devices have better energy recovery efficiency and higher output voltage for the same size. However, their disadvantage is that they require external power supplies to maintain the voltage of the two capacitive substrates during operation and cannot cause a short circuit when used [23]. Compared with electromagnetic and electrostatic energy harvesting devices, piezoelectric energy harvesting devices have a simple structure and high output voltage [24,25]. The typical structure of a piezoelectric energy harvester consists of a cantilever beam with a mass block at the free end that converts mechanical energy into electrical energy at resonant frequencies, but the piezoelectric effect is not satisfactory when the vibration amplitude is low.

However, the upper limit of efficiency of a single electromechanical system for power generation is low, so adding multiple mechanisms in the device is an effective way to increase the output power [26]. Currently, more and more scholars are using multiple mechanisms for power generation, such as piezoelectric and electrostatic [27], electromagnetic and frictional electric [28], etc. After much research, it has been proven that piezoelectric energy harvesters have large output voltage due to their high resistance, but the output current is small, from a few microamps to tens of microamps, which is suitable for working in a high load environment, as shown in [15,29]. However, electromagnetic energy harvesters have large output current and small output voltage, from tens of millivolts to hundreds of millivolts, which cannot meet the general requirements of the rated voltage of devices and is suitable for a smaller load environment [2,30,31]. The two mechanisms can perfectly complement each other, so in recent years, many scholars have combined them and proposed many new structures of vibration energy harvesters.

**Figure 1:**
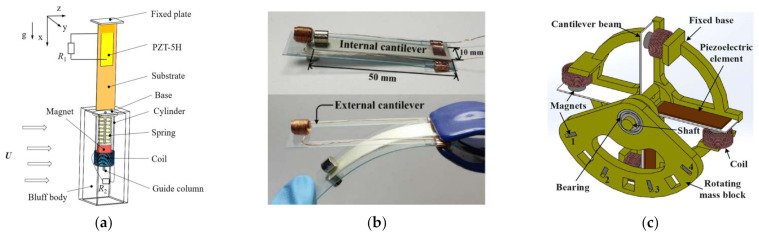
Various structures of energy harvester: (**a**) a piezoelectric–electromagnetic energy harvester with internally placed coils; (**b**) a piezoelectric–electromagnetic hybrid energy harvester based on frequency up-conversion; (**c**) a multimodal piezoelectric–electromagnetic hybrid energy harvester.

Challa [32] et al. proposed a cantilever beam structure piezoelectric–electromagnetic hybrid energy harvester that consisted of a piezoelectric cantilever beam, a permanent magnet (as an end mass block), and an induction coil. The maximum output power of the hybrid device was experimentally measured to be 332 µW at a resonant frequency of 21.6 Hz, while the optimized maximum output powers of piezoelectric and electromagnetic generation alone were 257 µW and 244 µW, respectively. Karami [33] et al. proposed a novel piezoelectric–electromagnetic hybrid nonlinear energy harvester that used two mutually repulsive magnets to generate nonlinear magnetic force, which broadened the frequency band of the harvester and improved the power generation performance. Mahmoudi [34] et al. combined a piezoelectric solid-supported beam and magnetic levitation structure to design a piezoelectric–electromagnetic hybrid energy harvester with nonlinear stiffness. Compared with a pure magnetically levitated energy harvester, with a nonlinear elastic guide, the power density and frequency bandwidth of the hybrid magnetically levitated energy harvester were increased by 60% and 29%, respectively. Li et al. [35] designed a piezoelectric–electromagnetic energy harvester with internally placed coils. Its energy generation principle was based on the mass block affected by the wind speed. It consisted of a beating piezoelectric energy harvester and an electromagnetic energy harvester (Figure 1a), which was a piezoelectric sheet attached at the fixed end of the cantilever beam and a mass block fixed at the free end. When the wind passed through the mass block, the pneumatic damping caused by the flow separation caused the cantilever beam to destabilize and generate periodic oscillation, and the piezoelectric power output and electromagnetic power output (produced by cutting the magnetic induction line) were caused at the same time. The effective output power of this harvester was 3.57 mW when the wind speed was 9 m/s, and that of a classical sprint piezoelectric energy harvester without a magnet reached 1.68 mW. Thus, the hybrid harvester output 112.5% more effective power.

The above various piezoelectric–electromagnetic hybrid harvesters had a limitation: the vibration frequency of nature is generally low (0–20 Hz), while the vibration response frequency of most of the aforementioned energy harvesters was particularly high, so they would not be effective in practical application for power generation. The current main techniques for energy harvesting in low-frequency environments are frequency up-conversion [26,36,37] and multistability [38]. Soonjae Pyo [26] et al. designed a new up-conversion piezoelectric–electromagnetic hybrid energy harvester consisting of two cantilevers and piezoelectric materials, as shown in Figure 1b. Two cantilevers were prepared from PVC (polyvinyl chloride). The inner cantilever was pasted with different sizes of PVDF (polyvinylidene fluoride), and a permanent magnet was fixed at the end. The outer cantilever was fixed with a coil of 100 turns. The device captured an open-circuit peak voltage of 263 V at a piezoelectric vibration frequency of 31 Hz and generated a maximum power of 3.16 mW at a 6 MΩ load resistance, while the electromagnetic part reached a peak voltage of 106 mV at 62 Hz with an output power of 2.60 mW. The total output power was 5.76 mW. At the ultralow frequency of 0.5–2 Hz, it could maintain the internal frequency of 26 Hz through up-frequency conversion technology to maintain power-generation efficiency. Ge [38] et al. proposed a multimodal hybrid energy harvester as shown in Figure 1c. It was a multidirectional human motion vibration energy harvester using piezoelectric and electromagnetic transduction techniques. Four piezoelectric and electromagnetic coupled cantilever beams were mounted on an annulus. A second rotating body circular block was mounted on a bearing and was free to rotate after installation. When the circular mass block was excited, it rotated at low frequency like a pendulum; in turn, the magnet mounted on the cantilever moved as a response to the block magnet. The cantilever beam was excited by this interaction and vibrated at its own higher frequency (42 Hz), so that both the piezoelectric and electromagnetic components gained energy. At a frequency of 4.8 Hz and an acceleration of 0.8 g, the power generated by the single piezoelectric was 1.28 mW, and that generated by the single electromagnetic was 0.03 mW.

Although the above hybrid energy harvesters can be adapted to low-frequency environments, they have some drawbacks. For example, the direction of energy capture is too singular to capture energy for multidirectional vibrations, resulting in low energy capture efficiency. The permanent magnets restrict the oscillation amplitude of the piezoelectric beam, resulting in low piezoelectric output power, etc. Based on this, this paper proposes a multistable magnetically coupled ring-shaped piezoelectric–electromagnetic hybrid energy harvester that consists of two parts: piezoelectric and electromagnetic energy harvesting structures. We first established an analytical model and combined the piezoelectric drive principle and electromagnetic drive principle to build an equivalent circuit diagram and analyze the theoretical output power. Then, we fabricated a physical hybrid energy harvester and built a vibration experimental system. A test compared the output voltage and output power of a non-magnetically coupled piezoelectric harvester, a magnetically coupled piezoelectric harvester, and the magnetically coupled piezoelectric–electromagnetic hybrid harvester. The experiments proved that the piezoelectric–electromagnetic hybrid harvester could simultaneously complete energy harvesting in two directions and output higher power under lower-frequency ambient vibration.

## 2. Design of Energy Harvester

**Figure 2:**
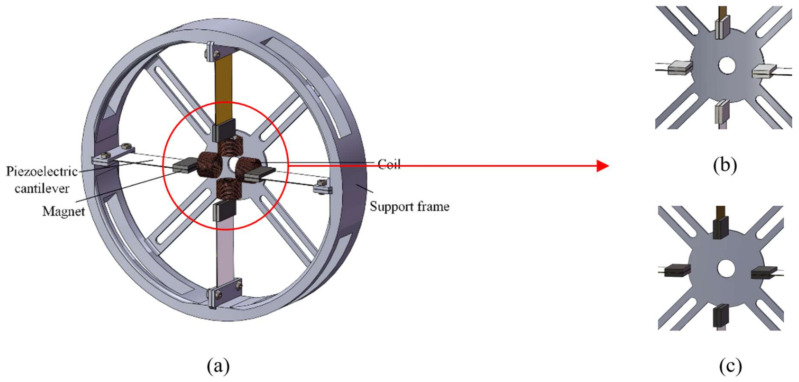
Schematic diagram of the structure of the energy harvester: (**a**) a schematic diagram of the cross-magnetically coupled piezoelectric–electromagnetic energy harvester; (**b**) a schematic diagram of the fixed permanent magnet at the end of the cantilever beam; (**c**) a schematic diagram of the fixed iron block at the end of the cantilever beam.

**Figure 3:**
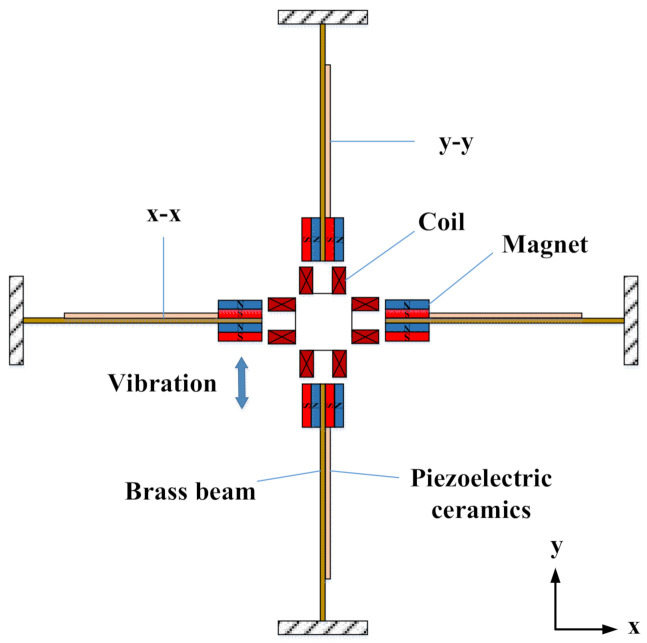
Schematic diagram of the piezoelectric–electromagnetic coupling cantilever beam.

As shown in Figure 2a, the harvester consisted of two parts: a piezoelectric system and an electromagnetic system. In the piezoelectric part, four cantilever beams of identical size were fixed symmetrically on the inner side of the ring support frame through the clamping structure. The material of the cantilever beams was brass. A piezoelectric ceramic sheet made of PZT-5H was pasted on the brass cantilever beams. NdFeB permanent magnets were fixed at the end of the brass cantilever beams and were adsorbed on both sides of the cantilever beams. There were four groups of piezoelectric power generation structures. They were fixed at an angle of 90 degrees between two and two in the clamping structure inside the ring-shaped support frame, and the permanent magnets at the end of the cantilever beam were in the same magnetic pole direction at the relative positions (the magnetic poles were positioned as in Figure 3). In the electromagnetic part, a circular abutment was attached to the inside of the ring support frame, and four coils of identical size and turns were fixed to the circular abutment in a cruciform symmetry, forming an electromagnetic energy harvest system with four permanent magnets. Compared with the conventional design, where in the permanent magnets pass through the inside of the coils, the magnetic flux was reduced by placing the coils in this way, but this did not impede the motion of the cantilever beam. In subsequent experiments, we compared the piezoelectric module generation capability of the energy harvester with permanent magnets fixed at the end of the cantilever beam (Figure 2b) and with iron blocks of the same size and weight fixed at the end (Figure 2c), and it was clear that the permanent magnets interacted with each other and affected the motion of the cantilever beam.

## 3. Operating Principle

The electric energy generated by this hybrid energy harvester could be obtained in two ways. One was the piezoelectric effect. Environmental vibration gives an acceleration to the cantilever beam, which deforms the piezoelectric ceramic on the cantilever beam and generates an electric potential to emit a voltage. The other was the electromagnetic effect, which is based on the electromagnetic induction of Faraday’s law. Current is generated by a permanent magnet vibrating continuously to cut the magnetic induction lines of the coil. The magnitude of the current is determined by the number of turns of the coil, the distance between the permanent magnet and the coil, and the rate at which the permanent magnet cuts the magnetic induction lines.

The piezoelectric structure could collect vibration energy in all directions along the xy plane. As shown in Figure 3, when the device was subjected to vibration along the y-axis, the permanent magnets on the x-x cantilever beam vibrated up and down, resulting in deformation of the piezoelectric ceramics on the surface of the cantilever beam and output voltage. Under the action of attraction and repulsion between magnets, the permanent magnets on the y-y cantilever beam also produced left and right vibration and output voltage. Similarly, when the device was subjected to the vibration along the x-axis, the permanent magnet on the y-y cantilever beam vibrated left and right, resulting in the deformation of the piezoelectric ceramics on the surface of the cantilever beam and the output voltage. Under the action of attraction and repulsion between magnets, the permanent magnet on the x-x cantilever beam also produced periodic vibration and output voltage. This device could collect energy in both the x-axis and y-axis directions, and because of the special design of the ring structure, forces in any direction parallel to the ring surface could cause the cantilever beam to vibrate and thus generate a voltage. In the piezoelectric vibration, permanent magnets at the ends of the four cantilevers vibrated back and forth, and the coils cut the magnetic lines to generate electricity. The device could collect energy in two directions at the same time, and the combination of piezoelectric and electromagnetic energy capture methods improved the energy capture efficiency.

**Figure 4:**
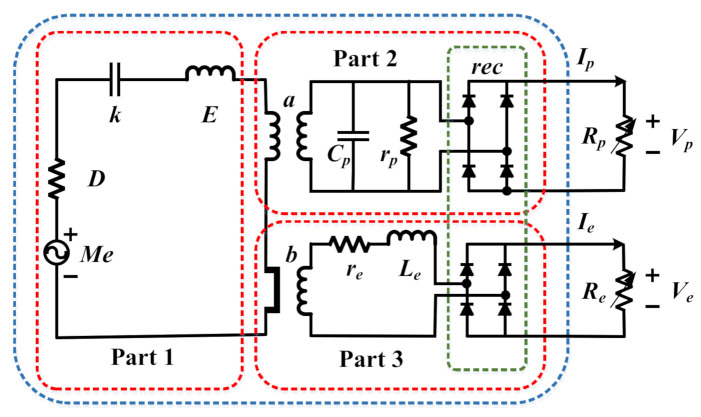
Piezoelectric–electromagnetic coupled equivalent circuit diagram of a single cantilever beam. Part 1 is the mechanical vibration module, part 2 is the piezoelectric module, and part 3 is the electromagnetic module.

Figure 4 shows an equivalent circuit diagram of a single piezoelectric–electromagnetic coupled cantilever beam. Part 1 denotes the mechanical vibration module: *Me* denotes the ambient vibration force, resistance *D* denotes the damping of the vibration process, capacitance *k* denotes the elastic potential energy of the cantilever beam, and inductance *E* denotes the kinetic energy generated in the vibration process. Part 2 denotes the piezoelectric module. The equivalent circuit of the piezoelectric module is coupled to that of the mechanical vibration module through the piezoelectric effect. *a* denotes the force-voltage coefficient, $C_{p}$ and $r_{p}$ are the corresponding clamp capacitance and internal resistance of the piezoelectric module, and $I_{p}$ and $V_{p}$ represent their corresponding output current and voltage when the external load resistance $R_{p}$ is connected. Part 3 represents the electromagnetic module. The equivalent circuit of the electromagnetic module is coupled to that of the mechanical vibration through the electromagnetic induction of the coil and permanent magnet. *b* represents the electromagnetic force-current coefficient, and $L_{e}$ and $r_{e}$ are the corresponding inductance and internal resistance of the electromagnetic coil. When the output port of the electromagnetic coil is connected to the external load resistance, $R_{e}$, $I_{e}$, and $V_{e}$ represent the corresponding output current and output voltage of the electromagnetic coil. *rec* represents the rectifier circuit, which is connected by four diodes. Because the diodes have the effect of single conduction, they can rectify the alternating current generated by the mechanical vibration into direct current for the load.

**Figure 5:**
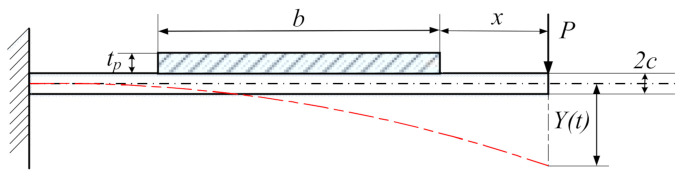
Deformation diagram of piezoelectric cantilever beam.

The piezoelectric energy harvester designed in this paper adopted a cantilever beam-type structure, and its composition included an elastic metal substrate (copper), a piezoelectric ceramic sheet (PZT-5H), and an end mass block (NdFeB). Figure 5 shows a force deformation diagram of the cantilever beam with piezoelectric sheet thickness $t_{p}$, cantilever beam thickness c and piezoelectric sheet width b.

The voltage generated on the surface of the piezoelectric sheet is related to the stresses applied to the material [39]:

(1) $$V\;=\;\frac{2d_{31}t_{p}\delta_{avg}}{\varepsilon }$$

where $t_{p}$ is the thickness of the piezoelectric ceramic (m), $d_{31}$ is the piezoelectric dielectric constant (C/N), $\delta_{avg}$ is the average stress on the cantilever beam (Pa), and ε is the piezoelectric ceramic dielectric constant (F/m).

At a distance *x* from the end of the cantilever beam, the bending moment applied to the cantilever beam is:

(2) $$M\left(x\right)\;=\;k_{1}Y\left(t\right)x$$

where $k_{1}$ is the cantilever beam’s equivalent stiffness and $Y\left(t\right)$ is the displacement of the free end of the cantilever beam relative to the fixed end.

The cantilever beam is subjected to the maximum stress at the free end. Assuming that the length of the cantilever beam is $L_{p}$, the moment of inertia of the cross-section of the cantilever beam I is:

(3) $$I\;=\;\frac{b{\left(2c\;+\;2t_{p}\right)}^{3}}{12}\;=\;\frac{2b\left(c\;+\;t_{p}{}^{3}\right)}{3}$$

where *b* is the width of the piezoelectric sheet and *c* is the thickness of the cantilever beam.

Thus, the average stress of the piezoelectric sheet is:

(4) $$\delta_{avg}\;=\;\frac{3k_{1}L_{p}\left(2c\;+\;t_{p}\right)}{8b{\left(c\;+\;t_{p}\right)}^{3}}Y\left(t\right)$$

Furthermore, the simplified open circuit voltage is:

(5) $$V\;=\;\alpha Y\left(t\right)$$

where α is the electromechanical coupling coefficient:

(6) $$\alpha \;=\;\frac{3k_{1}L_{p}d_{31}t_{p}\left(c\;+\;t_{p}\right)}{4\varepsilon b{\left(c\;+\;t_{p}\right)}^{3}}$$

If the load resistor resistance is $R_{L1}$, the piezoelectric output power is:

(7) $$p_{p}\;=\;\frac{\alpha^{2}R_{L1}Y_{0}}{{\left(R_{p}\;+\;R_{L1}\right)}^{2}}$$

where $Y_{0}$ is the root mean square value of 𝑌(𝑡) in N cycles.

According to Faraday’s law of electromagnetic induction, when the permanent magnet at the end of the cantilever beam cuts the magnetic field, the output power is [40]:

(8) $$P_{em}\;=\;\frac{{\left(NSB\right)}^{2}R_{e}\dot{H_{0}}}{{\left(r_{e}\;+\;R_{e}\right)}^{2}}$$

where N is the number of turns of the coil, S is the cross-sectional area of the coil, B is the magnetic induction strength of the permanent magnet, $R_{e}$ is the load resistance, $r_{e}$ is the coil internal resistance, and $\dot{H_{0}}$ is the root mean square value of $\dot{H}\left(t\right)$ in N cycles. $\dot{H}\left(t\right)$ represents the velocity of the magnet relative to the fixed end. The above equations provided the theoretical support for the design of our later test program. 

Table 1 shows the performance parameters of the selected piezoelectric material in this paper, PZT-5H.

## 4. Fabrication

**Figure 6:**
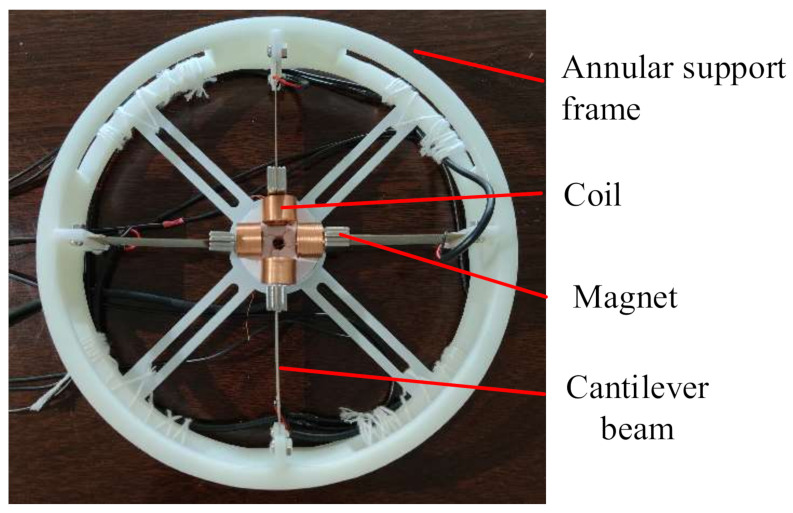
Physical diagram of the ring piezoelectric electromagnetic harvester.

The external annular support frame of the cross-shaped piezoelectric–electromagnetic harvester was constructed from resin material by 3D printing with an outer diameter of 175 mm and an inner diameter of 164 mm. Four clamping devices were symmetrically located at the top and bottom of the ring support frame, and there were 3 mm screw holes on the left and right sides of the clamping device. The copper substrate of the piezoelectric sheet was placed into the clamping device, and the piezoelectric sheet was clamped by two bolts (Φ3 mm × 6 mm). It was possible to change the length of the cantilever beam via the depth of the clamping position and thus change the inherent frequency of the cantilever beam. A circular abutment (Φ42 mm) in the middle of the ring support frame was used to fix four coils, and a small hole (Φ10 mm) in the middle facilitated the coil wiring passing through. The piezoelectric sheet was made of PZT-5H ceramic (40 mm × 20 mm × 0.2 mm) pasted on a copper substrate (60 mm × 20 mm × 0.2 mm) with a galvanized layer on the surface of the ceramic to prevent its oxidation. In the experiment, only one side of the cantilever beam was fixed with the piezoelectric ceramic, but in practice, both sides could be pasted with the piezoelectric ceramic in order to increase the power generation capacity. The permanent magnet fixed at the end of the cantilever beam was a NdFeB permanent magnet (20 mm × 10 mm × 2 mm). The inherent frequency of the harvester itself could be changed by adjusting the mass of the permanent magnet. Four coils (8.1 mm inner diameter, 14.5 mm outer diameter, 10 mm height) were fixed on the middle circular abutment, with 0.25 mm wire diameter, 500 turns, and 2 mm distance from the permanent magnet at the end of the cantilever beam. Theoretically, the closer the distance between the coil and the permanent magnet, the larger the magnetic flux in the coil and the stronger the electromagnetic power generation capability would be. However, in the actual test, a safety distance of 2 mm was set to prevent the coil from obstructing the movement of the cantilever beam. Table 2 shows the structural dimensions of the energy harvester, and Figure 6 shows the completed toroidal piezoelectric–electromagnetic energy harvester.

## 5. Experiments

**Figure 7:**
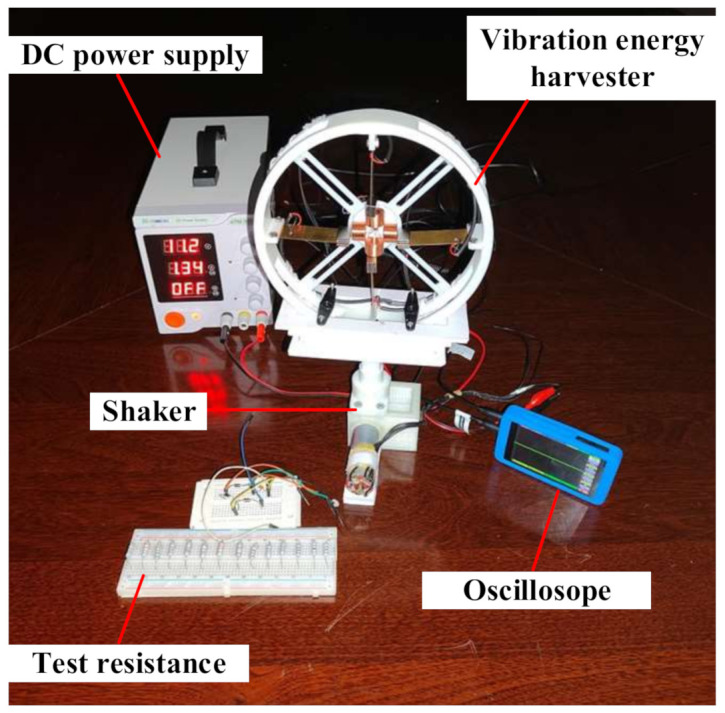
Vibration experimental table.

In order to test the performance of the vibration energy harvester designed in this paper, a vibration experimental table was set up to simulate the environmental vibration as shown in Figure 7. The table consisted of a shaker, an oscilloscope, a DC power supply, and a set of test resistances. The energy harvester was fixed on the shaker, and the vibration frequency was adjusted by changing the voltage of the DC power supply. The oscilloscope was used to observe the voltage signal changes (measurement accuracy to two valid digits after the decimal point), and the test resistances were used to test the optimum output power of the energy harvester. Open-circuit voltage tests for different masses at different frequencies, a multidirectional vibration test, output voltage and power tests for the non-magnetically coupled piezoelectric harvester and magnetically coupled piezoelectric harvester, and output voltage and power tests for the magnetically coupled hybrid harvester were carried out.

### 5.1. Open-Circuit Voltage Tests of Piezoelectric Harvester with Different Mass Blocks and Different Vibration Frequencies

**Figure 8:**
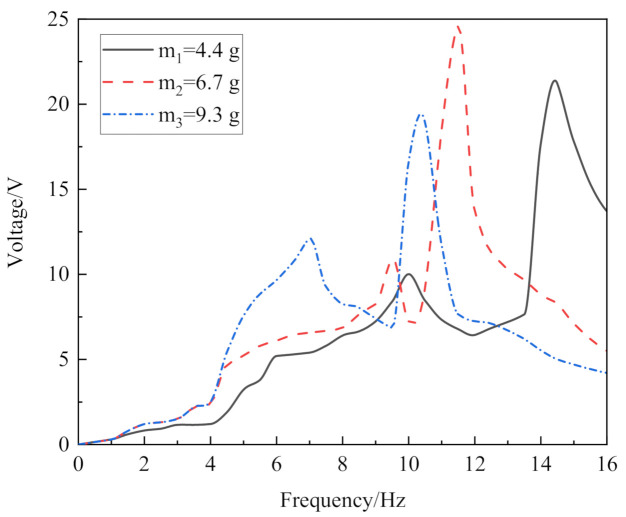
Open-circuit output voltage of different mass blocks at different frequencies. The voltage reached a maximum value of 24.6 V at m_2_ (6.7 g) and a vibration frequency of 11.5 Hz.

In order to test the relationship among the piezoelectric performance, the mass of the end mass block, and the excitation frequency, we measured the open-circuit voltage of the harvester at different mass blocks and different frequencies, as shown in Figure 8. An iron block with the same size as the permanent magnet (20 mm × 10 mm × 2 mm) was fixed at the end of the cantilever beam, and the acceleration was fixed. When the vibration of the energy harvester was at the resonant frequency point, the output voltage of the piezoelectric open circuit was the highest. 

When an iron block with a mass of 4.4 g was placed at the end of the cantilever beam, the resonant frequency point was 14.5 Hz, and the output voltage is 20.2 V. When the mass of the iron block was 6.7 g, the resonant frequency point was 11.5 Hz and the output voltage was 24.6 V. When the mass of the iron block was 9.3 g, the resonant frequency point was 10.5 Hz, and the output voltage was 18.9 V. There was a small peak before the resonant frequency due to the insufficient stiffness of the ring structure. Theoretically, when the mass of the block at the end of the piezoelectric cantilever beam is larger, the vibration amplitude of piezoelectric cantilever beam should larger, the deformation more obvious, and the output voltage higher. However, the deformation caused by excessive amplitude can also fracture the brittle piezoelectric ceramic. During the test, when the end iron block was 9.3 g, the copper substrate suffered a slight plastic deformation with the longer vibration time, indicating that the deformation recovery range of the copper substrate had been exceeded. Therefore, the end mass block of 6.7 g could ensure that the cantilever beam would work for a long time and obtain a good energy harvesting effect.

### 5.2. Open-Circuit Voltage Comparison of End Iron and End Magnet at Resonant Frequency and Multidirectional Vibration Test

**Figure 9:**
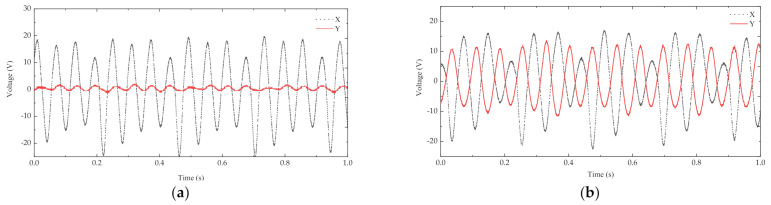
Open-circuit output voltage of piezoelectric harvesters: (**a**) the non-magnetically coupled piezoelectric harvester; (**b**) the magnetically coupled piezoelectric harvester.

The above experiment found the mass of the block corresponding to the optimum resonant frequency. An iron block of 6.7 g was placed at the end of the cantilever beam. When the iron block was excited by the resonant frequency of 11.5 Hz perpendicularly to the x-axis, the x-axis piezoelectric sheet produced a sinusoidal voltage waveform with a peak voltage of 24.6 V, while the y-axis piezoelectric sheet produced basically no voltage signal (Figure 9a). A 5.9 g NdFeb permanent magnet with the same size as the iron block was selected (NdFeb has a lower density than iron and thus a lighter weight with the same size). Then, the magnets interacted with each other. When excited by the resonant frequency of 11.5 Hz perpendicularly to the x-axis, both the x–x and y–y piezoelectric sheets produced regular sinusoidal voltage signals. The open-circuit output peak voltage of the x-x piezoelectric cantilever was 22.5 V, and that of the y-y cantilever was 13.4 V (Figure 9b). 

Although the peak voltage generated by the piezoelectricity of the x-x cantilever beam decreased under the influence of magnetic coupling, the magnet at the end of the y-y cantilever beam vibrated when it was affected by magnetic force, and the vertical vibration of the energy harvester amplified the vibration of the y-y cantilever beam to generate voltage. At the resonant frequency, the phase difference between the open-circuit voltages generated by the x- and y-axis piezoelectric plates was 0.04 s, which increased the voltage waveform density at the resonant frequency and realized multidirection energy harvest, significantly improving the energy harvest efficiency.

**Figure 10:**
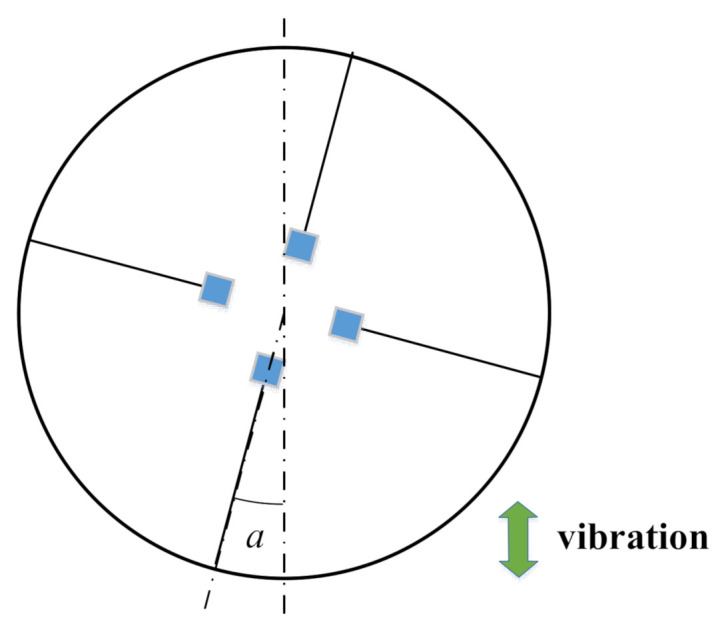
Schematic diagram of the vertical fixed radial deflection of the energy harvester.

**Figure 11:**
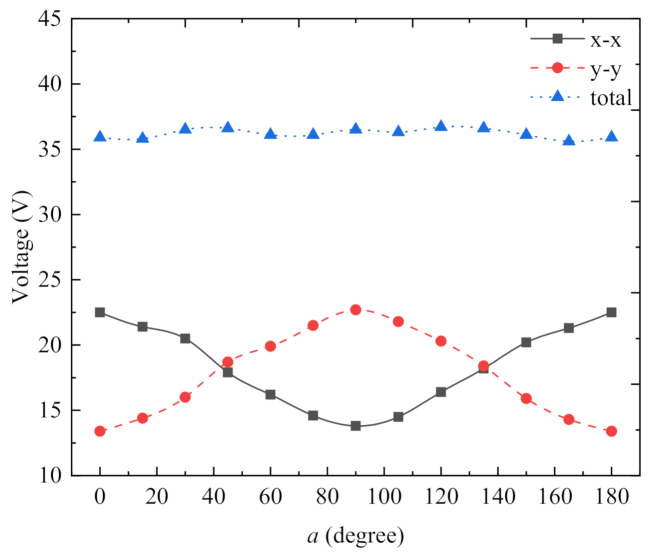
Output voltage for multidirectional vibration of the harvester.

Our proposed energy harvester could operate stably when subjected to vibration in any direction parallel to the ring surface, and the work efficiency remained basically stable. At the resonant frequency, we varied the radial angle of the energy harvester (Figure 10) and measured its open-circuit peak voltage at the angles of 0°, 15°, 30°, 45°, 60°, 75°, 90°, 105°, 120°, 135°, 150°, 165°, and 180° (Figure 11). Although the peak voltage values of the x-x-axis cantilever beam and the y-y-axis cantilever beam fluctuated significantly, the total voltage basically maintained a smooth curve. This indicates that the magnetically coupled piezoelectric energy harvester could maintain stability of power generation when subjected to environmental vibrations in any direction parallel to the ring surface.

### 5.3. Output Voltage and Power of Non-Magnetically Coupled and Magnetically–Piezoelectrically Coupled Energy Harvesters

**Figure 12:**
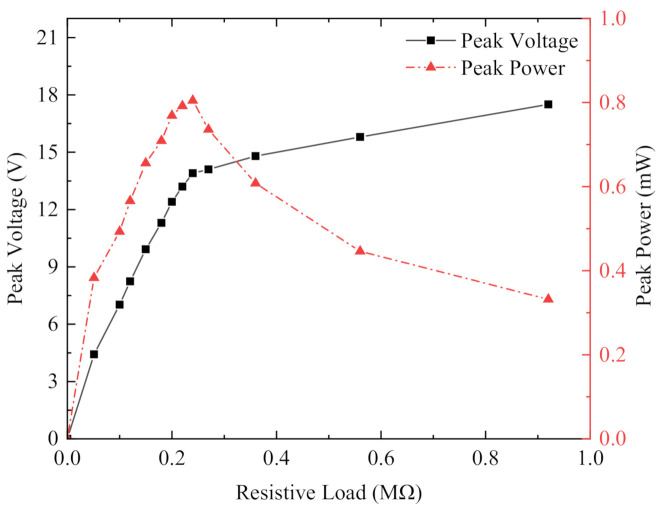
Output voltage and power of non-magnetically coupled piezoelectric capturer x-x piezoelectric sheet.

Output power was the most important index in the power generation performance experiment of the energy harvester. Because of the internal resistance of the piezoelectric sheet, the output power of the harvester changes with changes in load resistance. Figure 12shows the output voltage and output power of the non-magnetically coupled piezoelectric harvester at constant acceleration vibration of the x-x-axis cantilever beam with different external loads at a resonant frequency of 11.5 Hz. The mass block at the end of the cantilever beam was an iron block (6.7 g and the size is 20 mm × 10 mm × 2 mm). Since the piezoelectric energy harvester produced a sinusoidal alternating current and could not supply power to the load directly, each piezoelectric sheet was connected to the simplest standard energy recovery circuit for filtering and rectifying, converting the AC power to DC power, and then connected to resistors of different resistances for the best output power test. The output voltage generated by the x-x-axis piezoelectric sheet at resonant frequency increased as the load resistor resistance increased, while the output power first increased as the resistance value increased, then decreased slowly after reaching the maximum value, and finally tended to zero when the resistance value was extremely large. The load resistance of 240 kΩ was the best value to achieve the peak output power of 805 µW. The total output power was 1.61 mW, because there was a pair of x-x-axis piezoelectric sheets and the y-y-axis did not produce any output power.

**Figure 13:**
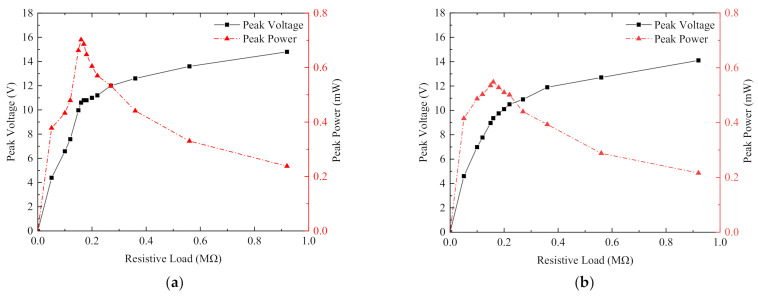
Output voltage and power of the magnetically coupled piezoelectric harvester: (**a**) load voltage and output power obtained from the rectifying circuit of x-x piezoelectric sheets; (**b**) load voltage and output power obtained from the rectifying circuit of y-y piezoelectric sheets.

Figure 13 shows the output voltage and output power of the magnetically coupled piezoelectric energy harvester at a resonant frequency of 11.5 Hz for each cantilever beam with different external loads. The mass block at the end of the cantilever beam was NdFeB, with a mass of 5.9 g and size of 20 mm × 10 mm × 2 mm. Figure 13a shows the load voltage and output power obtained from the rectification circuit connected to the x-axis piezoelectric sheet, and Figure 13b shows the load voltage and output power obtained from the rectification circuit connected to the y-axis piezoelectric sheet. As shown in Figure 13a the load resistor of 160 kΩ had the best resistance value to achieve the peak output power of 702 µW. The optimal load resistance in Figure 13b was also 160 kΩ, with a peak output power of 548 µW. The output power of the piezoelectric energy harvester was basically the same as that of the cantilever beam, so the total output power of the piezoelectric energy harvester was 2.5 mW. Compared with the non-magnetically coupled piezoelectric harvester, although the magnetic coupling led to a decrease in the output voltage of a single piezoelectric sheet, multidirection energy capture could be achieved under the single direction excitation, which increased the number of piezoelectric sheets involved in effective power generation and improved the total power.

### 5.4. Output Voltage and Output Power of Magnetically Coupled Piezoelectric–Electromagnetic Hybrid Energy Harvester

**Figure 14:**
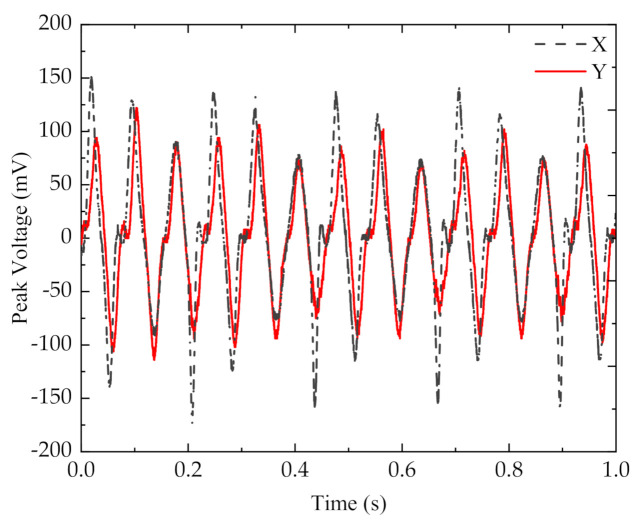
Electromagnetic energy harvester open-circuit output voltage.

According to Faraday’s law of electromagnetic induction, when a coil cuts a magnetic field, it generates current, the size of which is related to the number of turns of the coil, the cross-sectional area of the coil, the distance between the coil and the permanent magnet, the relative speed between the coil and the permanent magnet, etc. Here, coil had a diameter of 0.25 mm and 500 turns, and the distance between the coil and the permanent magnet at the end of the cantilever beam was 2 mm. In the energy harvester system, the piezoelectric structure and the electromagnetic structure shared a permanent magnet, so the electromagnetic vibration frequency was consistent with the piezoelectric vibration frequency. In the piezoelectric energy harvesting experiment, the output power was tested under the best resonant frequency of 11.5 Hz with different resistance values of external resistors. The maximum output power was obtained when the resistance value of the external resistance of the coil was equal to the internal resistance of the coil itself [38]. The coil internal resistance was 8.5 Ω. After testing, it was confirmed that the output power of external 8.5 Ω resistance was the largest. At this time, the output voltage of the coil opposite to the x-axis was 65.8 mV, and the output power is 509 µW. The output voltage of the coil opposite to the y-axis was 56 mV, and the output power was 369 µW, so the total electromagnetic output power was 1.76 mW at most. Figure 14 shows the open-circuit output voltage of electromagnetic generation.

**Figure 15:**
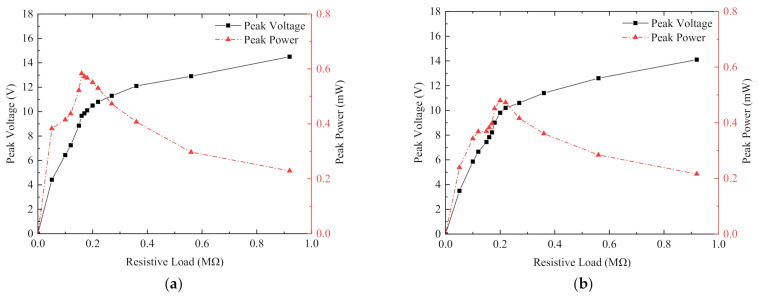
Magnetically coupled piezoelectric–electromagnetic energy harvester piezoelectric output voltage and power: (**a**) load voltage and output power obtained in the x-x piezoelectric sheet rectifier circuit; (**b**) load voltage and output power obtained in the y-y piezoelectric sheet rectifier circuit.

Figure 15 shows the output voltage and power of the piezoelectric module of the magnetically coupled piezoelectric–electromagnetic hybrid energy harvester. Compared with the magnetically coupled piezoelectric energy harvester with a single generating system, the coil obviously acted as a hindrance to the cantilever beam here. Figure 15a shows the load voltage and output power obtained in the x-x piezoelectric sheet rectifier circuit, and Figure 15b shows the load voltage and output power obtained in the y-y piezoelectric sheet rectifier circuit. As shown in Figure 15a, the load voltage increased as the resistance value of the load resistor increased and eventually tended to a stable value. The output voltage first increased as the resistance value decreased, then slowly decreased after reaching the maximum value, and finally tended to 0 when the resistance value reached the maximum value. The load resistance of 160 kΩ was the best resistance value, the peak output power is 583 µW. The optimum load resistance shown in Figure 15b was 200 kΩ, and the peak output power was 480 µW. The output power was basically the same as that of the cantilever beam, so the total output power of the piezoelectric energy harvester was 2.13 mW. Compared with the harvester without a coil, the total piezoelectric output power was reduced by 14.8%. This is because the electromagnetic coupling hindered the vibration of the piezoelectric sheet, so the total piezoelectric output power was reduced. However, the total power of the piezoelectric and electromagnetic energy harvester was 3.89 mW. Compared with the non-magnetically coupled piezoelectric harvester and the magnetically coupled piezoelectric harvester, the total power increased by 141.6% and 55.6%, respectively, which proved the effectiveness of the cross-magnetically coupled piezoelectric–electromagnetic hybrid energy harvester.

### 5.5. Experimental Conclusions

From the above experimental analysis, the piezoelectric–electromagnetic hybrid harvest energy structure reduced the output power compared with each independent harvest energy module because of the existence of mutual coupling force, which made the amplitude of the piezoelectric cantilever beam decrease. However, the total power of the hybrid was 3.89 mW, which was 55.6% higher than the output power of the independent piezoelectric output module and 121% higher than that of the independent electromagnetic module.

At present, some scholars have also proposed multidirectional energy harvesters, and the main characteristics of these are shown in Table 3 in comparison with this paper. The harvester proposed in [38] could collect energy in multiple directions using piezo-electromagnetic coupling; in that proposed in [41], piezoelectricity was used to collect energy in three directions. However, the energy these devices harvested was relatively low compared with that harvested by the energy harvester proposed in this paper.

## 6. Conclusions

In this paper, a cross-magnetically coupled piezoelectric–electromagnetic hybrid energy harvester is proposed. The principle of action and output power of the device were analyzed, and then a physical object was fabricated, a test rig built, and comparative tests conducted. The results showed that the device could obtain a high energy harvest effect under excitation in any direction parallel to the ring surface and that the required ambient excitation frequency was low, which made the energy harvest easier and more efficient. In addition, the test results showed that the hybrid energy harvester had a significant energy harvest effect compared with a non-magnetically coupled piezoelectric energy harvester and a magnetically coupled piezoelectric energy harvester; the output power increased by 141.6% and 55.6%, respectively.

## Figures and Tables

**Table 1:** Piezoelectric material performance table.

Piezoelectric Materials	PZT-5H
Electromechanical coupling factor	0.76
Relative permittivity	3400
Piezoelectric constant *d*_31_ (10^-12^ C/N)	2.75
Voltage constant (10^−3^ Vm/N)	9.3
Elastic modulus (10^9^ N/m^2^)	60.9

**Table 2:** Structural sizes and physical parameters.

Heading	Material	Size (mm)
Piezoelectric material	PZT-5H	40 × 20 × 0.2
Substrate	Copper	40 × 20 × 0.25
Magnet	NdFeB	20 × 10 × 2
Coil	Copper	Inner diameter 8.1, outer diameter 14.5, height 10
Support frame	UV Curable Resin	Inner diameter 164, outer diameter 175

**Table 3:** Comparison of several multidirection energy harvesters.

Study	Micromachines 2020 [38]	Rev. Sci. Instrum. 2021 [41]	This Paper
Structure	Complex	Simple	Simple
Directional of vibration	Multidirection vibration (any direction in surface)	Tridirectional vibration (specific)	Multidirection vibration (any direction in surface)
Energy harvesting method	Piezoelectromagnetic coupling	Piezoelectricity	Piezoelectromagneti ccoupling
Size	Outer diameter 70 mm	Length 80.4 mm, height 40 mm	Inner diameter 164 mm, outer diameter 175 mm
Optimal load resistance	100 kΩ (PE), 80 Ω (EM)	95 kΩ	160–200 kΩ (PE), 8.5 Ω (EM)
Maximum output voltage	6.7 V	13 V	14.5 V
Maximum output power	1.31 mW	306.94 μW	3.89 mW
